# Surgical repair of a right coronary aneurysm with a coronary artery fistula to the right atrium

**DOI:** 10.1093/jscr/rjab286

**Published:** 2021-07-08

**Authors:** Shigeki Yokoyama, Kanetsugu Nagao, Akihiko Higashida, Masaya Aoki, Shigeyuki Yamashita, Akio Yamashita, Toshio Doi, Kazuaki Fukahara, Naoki Yoshimura

**Affiliations:** Department of Surgery, Faculty of Medicine, University of Toyama, Toyama, Japan; Department of Surgery, Faculty of Medicine, University of Toyama, Toyama, Japan; Department of Surgery, Faculty of Medicine, University of Toyama, Toyama, Japan; Department of Surgery, Faculty of Medicine, University of Toyama, Toyama, Japan; Department of Surgery, Faculty of Medicine, University of Toyama, Toyama, Japan; Department of Surgery, Faculty of Medicine, University of Toyama, Toyama, Japan; Department of Surgery, Faculty of Medicine, University of Toyama, Toyama, Japan; Department of Surgery, Faculty of Medicine, University of Toyama, Toyama, Japan; Department of Surgery, Faculty of Medicine, University of Toyama, Toyama, Japan

**Keywords:** coronary artery fistula, coronary artery aneurysm, surgical closure of coronary artery fistulas

## Abstract

A coronary artery fistula is a rare condition caused by abnormal coronary artery embryological development. Although most cases are asymptomatic, in some, the large shunt volume and the myocardial ischemia due to the steal phenomenon require surgical treatment. We present the case of a 40-year-old woman who presented with angina on exertion. Enhanced computed tomography showed a giant right coronary artery (RCA) aneurysm with an RCA-to-right atrium fistula. Because of the presence of symptoms and the presence of large fistulous tract, the patient was considered a surgical candidate. The procedure was performed under cardiopulmonary bypass. Ligation and closure of the fistula were performed in combination with dissection of the enlarged main trunk of the RCA and coronary artery bypass using the internal thoracic artery because of its potential for long-term patency. The postoperative course was uneventful.

## INTRODUCTION

A coronary artery fistula is a rare disease caused by an abnormality of the coronary artery embryological development [1]. Although most cases are asymptomatic, in some, the large shunt volume and the myocardial ischemia due to the steal phenomenon require surgical treatment [1–3]. Potential complications include coronary artery aneurysm rupture and infective endocarditis. [1, 4, 5]. Patients at risk for these fatal complications are candidates for surgical or transcatheter treatment. We report a case of a right coronary aneurysm with a coronary artery fistula to the right atrium.

## CASE REPORT

**Figure 1:**
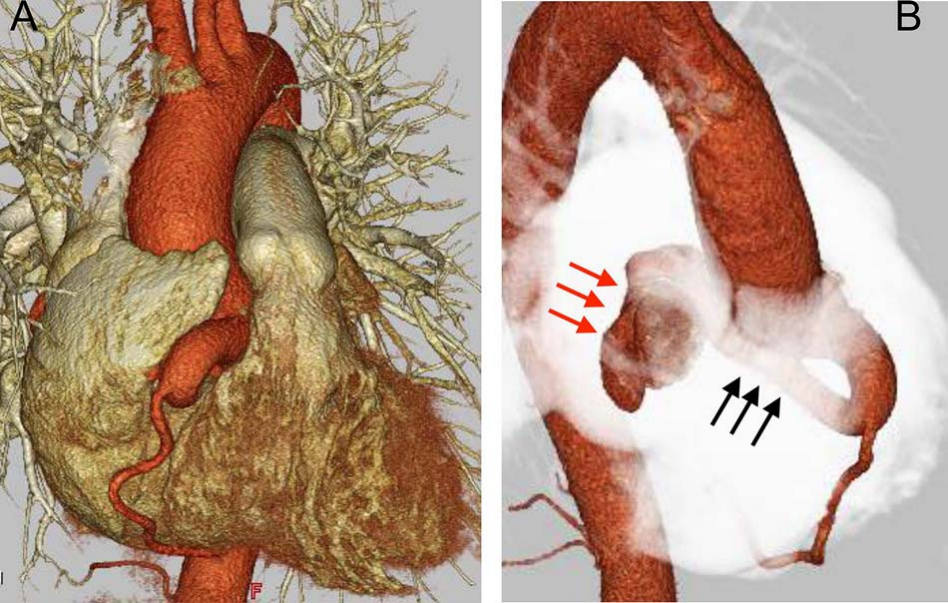
Preoperative CT findings; (**A**) frontal view; (**B**) upper right diagonal view; preoperation CT showed a dilated RCA (black arrow) with a diameter of 13.2 mm, and a giant aneurysm (red arrow) with an RCA-to-right atrium fistula.

The patient was a 40-year-old woman, who had been diagnosed with a continuous heart murmur since junior high school. After being asymptomatic for many years, she became aware of angina on exertion for which she visited her family doctor. Chest X-ray revealed an enlarged heart and mild pulmonary congestion. Serology revealed a mild elevation of brain natriuretic peptide (BNP), at 77.7 pg/ml. The electrocardiogram was normal. No ischemic changes were noted on treadmill stress electrocardiogram. Transthoracic echocardiography showed normal left ventricular motion and no valvular disease. In addition, cardiac Doppler ultrasound showed continuous turbulent flow into the right atrium. Enhanced computed tomography (CT) showed a significantly dilated (13.2 mm) right coronary artery (RCA) at its origin, with a coronary artery fistulous tract draining into the posterior aspect of the right atrium. This fistula formed a large mass (21 × 29 mm) on the dorsal surface of the superior vena cava. The diameter of the RCA, distal to the fistula, was normal (Fig. 1). On right heart catheterization, intracardiac pressure data were within normal limits, but an O2 step up was observed in the right atrium, and Qp/Qs was 1.3. She was diagnosed with a right coronary aneurysm with a coronary artery fistula to the right atrium. Because of worsening of her symptoms and the presence of a large fistulous tract, the patient was considered a surgical candidate.

**Figure 2:**
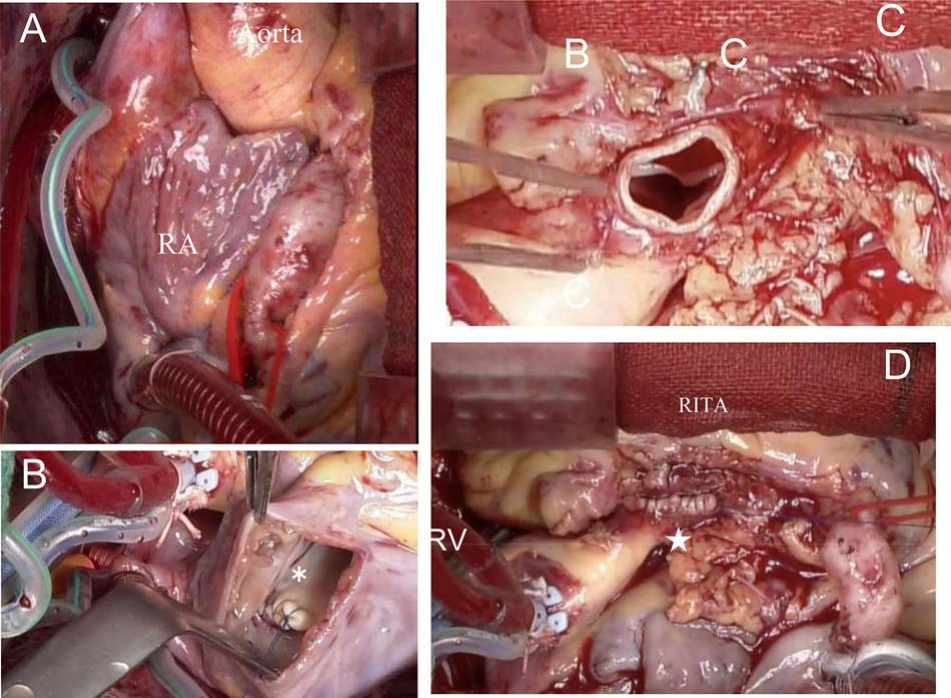
Intraoperative findings; (**A**) the RCA dilated from its origin; the coronary artery fistula is not visible because it is hidden by the right atrium; (**B**) the opening of the coronary artery fistula draining into the posterior wall of the right atrium, closed with 5-0 polypropylene continuous suture (*); (**C**) dissected origin of the dilated RCA; (**D**) dilated RCA closed with 4-0 polypropylene mattress with over and over suture to preserve the morphology of the sinus of Valsalva (★).

**Figure 3:**
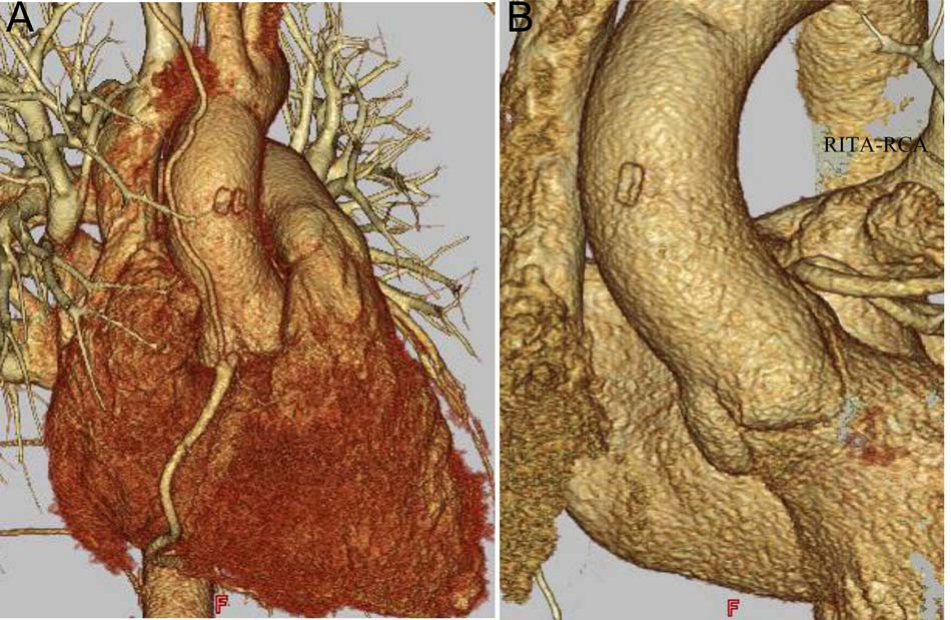
Postoperative CT findings; (**A**) RITA-RCA bypass; (**B**) the sinus of Valsalva is shown with preserved morphology; RITA, right internal thoracic artery.

The operation was performed via median sternotomy. The RCA was markedly enlarged from its origin, and the fistula branched from the middle toward the dorsal side of the superior vena cava (SVC). The RCA distal to the fistula had a normal diameter (Fig. 2A). After systemic heparinization, cardiopulmonary bypass (CPB) was established with ascending aortic cannulation, with venous drainage from bi-caval cannulation. The ascending aorta was cross-clamped and cardiac arrest was achieved with cardioplegic solution (CPS) infusion from the aortic root while compressing the coronary fistula. After snaring the SVC and inferior vena cava (IVC), the right atrium was opened. Inside the right atrium, a 9-mm tract opening draining dorsal to the SVC–right atrial junction was observed. The tract opening on the posterior wall of the right atrium was closed with 5-0 polypropylene continuous suture (Fig. 2B). The dilated origin of the RCA was resected, and the stump was closed with 4-0 polypropylene mattress with continuous over-and-over suture to preserve the morphology of the sinus of Valsalva. (Fig. 2D). The distal RCA was ligated at a normal diameter site and grafted to the right internal thoracic artery. The operative time was 230 min; the cardiopulmonary and cardiac arrest times were 118 and 58 min, respectively. Postoperative transthoracic echocardiography showed no residual shunt into the right atrium and no aortic regurgitation. Postoperative enhanced CT showed patency of right internal thoracic artery graft (Fig. 3A) and preserved morphology of the sinus of Valsalva (Fig. 3B). The postoperative course was good, and the patient was discharged uneventfully on the 16th postoperative day.

## DISCUSSION

We reported a case of a right coronary aneurysm with a coronary artery fistula to the right atrium. Coronary artery fistulas are congenital malformations of the coronary arteries during embryological cardiac development. Most cases are asymptomatic and discovered incidentally due to the more widespread use and improved performance of cardiac echocardiography and CT [6]. The left–right shunt volume may increase, resulting in congestive heart failure and pulmonary hypertension, and a coronary artery fistula can potentially steal blood from other branches of the artery [2, 3, 7]. Prior to the formation of coronary arteries, the ventricular wall is sinusoidal and directly perfused with blood from the ventricular cavity. As coronary arteries are formed from the epicardium, the myocardium becomes compacted. Coronary artery fistulas develop as a result of failure of myocardial compaction and persistent abnormal communication between the intramyocardial sinusoidal system and the coronary arteries. [8]. Coronary artery fistulas involve the right coronary arteries in about 55% cases and the left coronary arteries in 35% of cases. They drain into the right heart in 90% of cases [1]. Selective coronary angiography is the gold standard, but sometimes it is not possible to identify the exact location of the fistula [1]. In recent years, enhanced CT can be used as an adjunct to coronary angiography [6]. In this case, the RCA was significantly enlarged from its origin, and the contrast catheter could not wedge, resulting in poor vessel enhancement. The details of the coronary artery fistula, therefore, were not clarified by angiography. Previous literature suggests that surgical treatment be considered in patients with a shunt volume of more than 30–40%, pulmonary hypertension, heart failure, myocardial ischemia, history of bacterial endocarditis and aneurysms with a risk of rupture [9]. Aortic valve deformity associated with enlargement of the proximal coronary artery is also an indication for surgery [10]. Kamiya *et al*. [11] showed good surgical outcomes and reported that surgery should be performed to prevent ischemic heart disease even if the patient is asymptomatic. Transcatheter closure may be performed in cases of small coronary artery fistulas, but surgical treatment is preferred in cases presenting with complex branches or large aneurysms [12]. Although various techniques have been reported, the basic technique involves ligation and dissection of the fistula vessel under cardiac arrest using CPB, closure of the fistula from inside the intracardiac cavity and resection of the aneurysm [1]. In recent years, a transcatheter closure technique without CPB has been reported [13, 14]. Coronary artery fistulas have many variations depending on the case, and it is important to select the appropriate technique for each case. In this case, the RCA proximal to the fistula was markedly dilated. Therefore, we opted to perform a coronary artery bypass on the distal side after resecting the dilated area. For surgical closure of the dilated origin of the RCA, we initially considered a patch closure from within the aorta, but we ultimately opted for a simpler outside suture. The postoperative morphology of the sinus of Valsalva was excellent, and aortic regurgitation was not observed, suggesting that our surgical approach was appropriate. Given the patient’s young age, the internal thoracic artery was used as a graft because of its potential for long-term patency.

## CONFLICT OF INTEREST STATEMENT

None declared.

## FUNDING

None.
